# Do Organic Substrates Drive Microbial Community Interactions in Arctic Snow?

**DOI:** 10.3389/fmicb.2019.02492

**Published:** 2019-10-31

**Authors:** Benoît Bergk Pinto, Lorrie Maccario, Aurélien Dommergue, Timothy M. Vogel, Catherine Larose

**Affiliations:** ^1^Environmental Microbial Genomics, Laboratoire Ampère, École Centrale de Lyon, UMR CNRS 5005, Université de Lyon, Lyon, France; ^2^Univ Grenoble Alpes, CNRS, IRD, Grenoble INP, Institut des Géosciences de l’Environnement, Grenoble, France

**Keywords:** competition, cooperation, networks, snow, organic acids

## Abstract

The effect of nutrients on microbial interactions, including competition and collaboration, has mainly been studied in laboratories, but their potential application to complex ecosystems is unknown. Here, we examined the effect of changes in organic acids among other parameters on snow microbial communities *in situ* over 2 months. We compared snow bacterial communities from a low organic acid content period to that from a higher organic acid period. We hypothesized that an increase in organic acids would shift the dominant microbial interaction from collaboration to competition. To evaluate microbial interactions, we built taxonomic co-variance networks from OTUs obtained from 16S rRNA gene sequencing. In addition, we tracked marker genes of microbial cooperation (plasmid backbone genes) and competition (antibiotic resistance genes) across both sampling periods in metagenomes and metatranscriptomes. Our results showed a decrease in the average connectivity of the network during late spring compared to the early spring that we interpreted as a decrease of cooperation. This observation was strengthened by the significantly more abundant plasmid backbone genes in the metagenomes from the early spring. The modularity of the network from the late spring was also found to be higher than the one from the early spring, which is another possible indicator of increased competition. Antibiotic resistance genes were significantly more abundant in the late spring metagenomes. In addition, antibiotic resistance genes were also positively correlated to the organic acid concentration of the snow across both seasons. Snow organic acid content might be responsible for this change in bacterial interactions in the Arctic snow community.

## Introduction

Dynamic changes in nutrient concentrations have been shown to influence bacterial interactions with ramifications for microbial community structure and function (Friedman and Gore, 2017; Khan et al., 2018). In these pure culture studies, either cooperation or competition was the dominant interaction strategy depending on the nutrients considered and their concentrations (Brockhurst et al., 2008, 2010, Lambert et al., 2011, 2014; Ravindran, 2017). Interference competition was hypothesized to be mediated by antibiotic release (Cornforth and Foster, 2013; Oliveira et al., 2015; Ponce-Soto et al., 2015; Song et al., 2017) and was shown to be affected by the nutrient supply (Hol et al., 2014). For example, a sensitive *Escherichia coli* strain was observed to co-exist with a colicin-secreting *E. coli* strain when co-cultivated on a poor growth medium (sugars), but not on a rich medium (amino acids and peptides), where the colicin-secreting *E. coli* strain released antibiotics (Hol et al., 2014). Cooperation was also proposed to be mediated by either metabolic or genetic exchanges between different collaborative strains (Nogueira et al., 2009; Mc Ginty et al., 2011; Dimitriu et al., 2014, 2015; Benomar et al., 2015; Wall, 2016; Tecon and Or, 2017) and has also been shown to be affected by nutrient supply (Benomar et al., 2015). Several studies have examined the importance of horizontal gene transfer in maintaining cooperation in synthetic bacterial communities (Czárán and Hoekstra, 2009; Nogueira et al., 2009; Dimitriu et al., 2014, 2015; Wall, 2016). Therefore, cooperation might be promoted by increasing assortment among cooperative alleles (Dimitriu et al., 2014) or by increasing kin selection (Nogueira et al., 2009; Wall, 2016). In addition, most of the genes coding for public goods appeared to be preferentially localized on mobile genetic elements (plasmids) and at hotspots of genome recombination (Nogueira et al., 2009).

The majority of research concerning nutrient-related effects on bacterial interactions has been generated with culture-based experiments (Mitri and Foster, 2013). While these studies have provided information on different nutrient effects on bacterial interactions under controlled conditions, they might not predict microbial interactions in the environment. Microcosm or mesocosm approaches have been used more recently to study microbial communities and the results have varied (Ponce-Soto et al., 2015; Ali et al., 2016; Song et al., 2017). Although no studies concerning the effect of carbon content on microbial interactions have been published to date, one study measured an increase in antibiotic resistance genes in strains of *Enterococcus faecalis* cultivated in eutrophic sediment mesocosms amended with nitrogen and phosphorus (Ali et al., 2016). Other studies observed a decline of antibiotic resistance in cultivable bacterial populations from an oligotrophic lake in mesocosms amended with nitrogen and phosphorus and from soil bacteria cultivated on agar plates amended with increasing nutrient medium concentrations (Ponce-Soto et al., 2015; Song et al., 2017). The main difference between these two sets of studies is that one used a PCR based method to track antibiotic resistance (Ali et al., 2016), while the others used culture based methods (Ponce-Soto et al., 2015; Song et al., 2017). Culture based techniques could have a higher bias since they alter the bacterial community by selecting members able to grow on media.

Nutrient dynamics also affect bacterial community structure (Campbell et al., 2010). For example, an increase in organic matter during soil fertilization was shown to decrease bacterial community evenness in Arctic tundra soil (Koyama et al., 2014). The observed effect of nutrients on bacterial community structure might be indirect and mediated in part by bacterial interactions. The low cultivability associated with environmental bacteria might be mainly due to the co-dependency of bacteria that are auxotrophic for some critical functions and, therefore, are obligate co-operators (Pande and Kost, 2017). Thus, bacterial communities might be viewed as networks of cooperating and competing individuals. Such a view has been explored by recent experiments that show a differential growth rate of environmental bacterial strains when co-cultured with other specific strains (Pande et al., 2014; Ren et al., 2015; Vartoukian et al., 2016). Bacterial interactions could provide a selective advantage to bacterial species as a function of nutrient concentrations and subsequently influence bacterial community structure.

Tracking bacterial interactions *in situ* can be performed through networks, such as co-variance networks based on taxonomic data (Faust and Raes, 2012). This approach has been used for microbial communities from oceans (Ruan, 2006; Lima-Mendez et al., 2015), soils (Barberán et al., 2012; Ding et al., 2015), human microbiomes (Faust et al., 2012) and heavy-metal-polluted sediments (Yin et al., 2015). These networks often use co-variance to infer positive (cooperative) and negative (competitive) bacterial interactions (e.g., Ruan, 2006), but co-variance might also indicate that the populations are responding to other stimuli, simultaneously. An approach combining gene markers for bacterial interactions based on pure culture studies and taxonomy-based co-variance networks described above should strengthen the results obtained. Here, we applied this combined approach using antibiotic resistance as the surrogate for competition and plasmid structural genes for collaboration, and taxonomy-based co-variance networks on microbial communities sampled from an Arctic snowpack over the spring season. Arctic snow microbial communities were selected because arctic snow carbon content varies by several orders of magnitude during the spring season (Twickler et al., 1986) and is generally considered a low carbon environment. Recently, using COG functions characteristic of oligotrophy or copiotrophy as proposed by Lauro et al. (2009), Maccario et al. (2019) showed that arctic snow bacterial communities were adapted to oligotrophic lifestyles. However, oligotrophic the arctic snow environment is, carbon content increases over the spring season (Hacking et al., 1983; Twickler et al., 1986; Haan et al., 2001; Grannas et al., 2007). In addition, Arctic snow has varying nutrient conditions that affect bacterial community structure and function (Larose et al., 2013). We hypothesized that increases in organic acids (as a soluble subset of potential organic substrates) in the warming spring snow would increase competition (and reduce collaboration).

## Materials and Methods

### Field Sampling

Snow samples were collected during a 2011 springtime field campaign in Ny-Ålesund (Svalbard, Norway, 78°56′N, 11°52′E). Surface snow layers (upper 3 cm) (2L meltwater equivalent) were collected into sterile bags using a sterilized shovel as described previously (Larose et al., 2010a). A total of 31 samples were collected between mid-April to beginning of June 2011. The spring research campaign was held between April, 2011 and June, 2011 at Ny Ålesund in the Spitsbergen Island of Svalbard, Norway (78°56′N, 11°52′E). The field site, a 50 m^2^ perimeter with restricted access (to reduce contamination from human sources), is located along the south coast of the Kongsfjorden, which is oriented SE-NW and open to the sea on the west side (Supplementary Figure S1). We added a map in supporting information. The Kongsfjorden was free of sea ice throughout the campaign. Specific sampling dates can be found in the chemistry table (see dataset at Supplementary Table S1). In addition, different weather and snow conditions were monitored over the sampling period (Supplementary Figure S2). Samples for snow chemistry were collected, stored frozen, sent back to the laboratory in France for analysis as described in Larose et al. (2010a, b). Snow samples collected for microbiology were processed immediately after collection in the field laboratory. Samples were left to melt at room temperature prior to filtering onto sterile 0.22 μM 47 mm filters (Millipore) using a sterile filtration unit (Nalge Nunc International Corporation) and filters were stored in Eppendorf tubes filled with the extraction buffer from the PowerWater extraction kit (MoBio) at −20°C for further analysis. Samples for major ions and particles were collected in sterile polycarbonate Accuvettes© sealed with polyethylene caps. All samples were stored frozen (−20°C) and in the dark until analysis.

### Chemical Analysis

Samples were melted in a class 100 clean room at LGGE-CNRS laboratory (Grenoble, France). They were then transferred into Dionex glass vials previously rinsed with ultra-pure Millipore water (conductivity > 18.2 mΩ, TOC < 10 ng/g) and analyzed less than 24 h after melting. Analyses were performed by conductivity-suppressed ion chromatography using a Dionex ICS 3000© apparatus and a Dionex AS40© autosampler placed in the clean room facilities. Different chemical parameters were measured during this study (e.g., major/minor ions, organic acids, and pH). Soluble anions (methyl sulfonic acid (MSA), SO_4_, NO_3_, Cl) and cations (Na, NH_4_, K, Mg, Ca) and organic acids were analyzed by ionic chromatography (IC, Dionex ICS3000). AS/AG 11HC and CS/CG 12A columns were used for anions and cations analyses, respectively. All chemical analyses were carried out at on the airOsol platform of the IGE laboratory in Grenoble, France. This data set can be found in Supplementary Table S1. The following parameters were used for statistical analyses [Organic acids (oxalate, lactate, glutarate, propionate, succinate, formate, acetate), NO_3_^–^, NH_4_^+^, SO_4_^2–^, mercury, fluoride, calcium, magnesium, bromide, strontium, lithium, sodium, chloride, potassium, number of particles, methyl sulfonic acid (MSA)] and pH. For values below the detection limit, we used the detection limit divided by 2.

### DNA Extraction and Sequencing

The DNA from 20 surface snow samples collected between April and May 2011 (CH3N-1 to CH3N-37 or early spring ES) and 16 surface snow samples collected from May to June 2011 (CH3N-40 to CH3N-76 or late spring LS) were extracted for taxonomic analysis. Snow was melted at 4°C before filtering on 0.2 μm filters. DNA was extracted from filters using the DNeasy PowerWater Kit (Qiagen) following the manufacturer’s instructions. Then, the DNA was quantified using the Qubit^TM^ dsDNA HS Assay Kit (Thermo Fisher Scientific) and the V3–V4 regions of the 16S rRNA genes were amplified by a PCR of 35 cycles at 92°C 30 s, 55°C 30 s and 72°C. air Forward primer is composed of the Illumina adapter 5′TC GTCGGCAGCGTCAGATGTGTATAAGAGACAG coupled to the 16s rRNA gene primer part CCTACGGGNGGCWGCAG and Reverse primer is composed of the Illumina adapter 5′GT CTCGTGGGCTCGGAGATGTGTATAAGAGACAG coupled to the 16s rRNA gene primer part GACTACHVGGGTAT CTAATCC. The 16S rRNA gene primers are from Klindworth et al. (2013). Simultaneous adapter insertion and amplification was performed using the Platinum PCR SuperMix (Invitrogen). Libraries for 16S rRNA gene sequencing were prepared using the 16S rRNA gene Library Preparation Workflow recommended by Illumina. Paired end sequencing was then carried out on a MiSeq sequencer (Illumina) at the laboratory in Lyon. Size of samples before and after clustering is provided in Supplementary Table S2.

Eight samples (CH3N−1 to CH3N−10) collected between April and May and twelve samples (CH3N-40 to CH3N-66) collected between May and June underwent metagenomic and metatranscriptomic sequencing. Not all samples were analyzed for 16S rRNA genes as some of the metagenomic samples did not have any DNA remaining for the 16S rRNA analysis. In addition, we selected extra samples for the 16S rRNA gene based network analysis. For the metatranscriptomic/metagenomic analyses, total nucleic acids were extracted using PowerWater RNA isolation kit (MoBio) following the manufacturer’s instructions, except that the DNAse treatment step was omitted. The RNA fraction of nucleic acids was then further purified using RNeasy kit from Qiagen following the manufacturer’s instructions. cDNA libraries were prepared from RNA using Tetro cDNA synthesis kit (Bioline). DNA and cDNA samples were then amplified using multiple displacement amplification with the illustra^TM^ GenomiPhi^TM^ HS DNA Amplification Kit (GE Healthcare) since concentrations were too low for library preparation and sequenced using a Roche 454 Titanium pyrosequencer to generate longer reads than illumina MiSeq. Not all samples had sufficient amounts of DNA for sequencing, resulting unbalanced groups (i.e., 8 for ES and 12 for LS). The reads produced from the 454 were 350 bp ± 100 bp average fragment length following quality filtering (Supplementary Figure S3). The depth of sequencing for each sample is reported in Supplementary Table S3. Sequences are publically available at ftp://ftp-adn.ec-lyon.fr/Snow_organic_acids_bacterial_interactions.

### Bioinformatic Pipeline for Quality Filtering, *de novo* Clustering, and 16S rRNA Gene Annotation

We used USEARCH (v 9.2) and the UPARSE pipeline (Edgar, 2013) for quality filtering and clustering of our 16S rRNA gene datasets (for details on parameters used see Supplementary Material and also the provided script). We annotated the representative sequence of each cluster using RDP classifier (Wang et al., 2007) with a bootstrap threshold of 80%. We normalized the OTU counts by using the R package MetagenomeSeq (Paulson et al., 2013).

### Metagenomic and Metatranscriptomic Annotation and Dataset Generation

The raw files from 454 pyrosequencing were processed using Mothur (Schloss et al., 2009) for quality filtering with the settings recommended in Schloss et al. (2011). FastQC (Andrews, 2010) was also used to control for base overrepresentation. Some remains of adapters were found and Usearch (Edgar, 2010) was used to trim our sequences. The resulting.fastq files were functionally annotated using EggNOG-Mapper (Huerta-Cepas et al., 2017), based on eggNOG orthology data (Huerta-Cepas et al., 2016), using the default parameters. The sequence searches were performed using diamond (Buchfink et al., 2015). Resulting annotations were imported into R (R Development Core Team, 2011) to build gene count tables. Reads annotated as eukaryotic sequences were filtered out based on the tax id associated to each sequence annotation using the R package taxize to obtain a bacterial and archaeal dataset (Chamberlain and Szöcs, 2013). The “Retrieve/ID mapping” function^1^ from uniprot was used to convert the string ids (EggNOG) into uniprot protein names to generate functional gene tables for each metagenomic and metatranscriptomic dataset. The GO annotation associated to these protein names was used for subsequent analyses.

### Chemical/Molecular Biology Data Analysis

The chemical data were evaluated for differences between sample groups. Data were log transformed (except pH) and a PCA was calculated using the ade4 package (Dray and Dufour, 2007) in R. Co-inertia analysis (Dolédec and Chessel, 1994) was used to test the impact of snow chemistry on bacterial communities using the R package ade4 (Dray and Dufour, 2007). Chemical data sets were compared to microbial taxonomy (OTU table 16S rRNA gene at the genus level), metagenomes (gene annotation level and EggNOG-Mapper annotations) and metatranscriptomes (gene annotation level and EggNOG-Mapper annotations). The significance of each co-inertia was tested using a permutation test (10000 permutations).

### ANOSIM Analysis

The OTU tables were processed with the ADONIS function from the vegan (Dixon, 2003) package in R to carry out ANOSIM (ANalysis Of SIMilarities) analysis. This is a non-parametric test to detect whether more similarities exist between samples inside a sampling group than with the rest of the dataset. We used this method with a randomization test (10000 permutations) to test for differences in similarity between the groups of samples from early spring (ES) and late spring (LS).

### Network Analysis With the OTUs

Based on the OTU tables generated previously with USEARCH for ES and LS groups, a co-variance network was built. Prior to building the network, a filtering step was used to remove OTUs present in less than eight samples (50% of the samples used to build each network). FastLSA (Durno et al., 2013), an improved version of LSA (Local Similarity Analysis) (Ruan, 2006) was used to compute the networks. LSA has been shown by Weiss et al. (2016) to detect significant co-variance on time series data. We used a lag of zero and filtered out the results that were not significant at the 95% confidence interval (*p*-val < 0.05). These data were then imported into R and the packages igraph (Csardi and Nepusz, 2006) and GGally, which is an extension from ggplot2 (Wickham, 2009), were used to visualize the co-variance networks obtained. After the network assembly, we compared their respective densities.

### Functional Analysis of Microbial Communities

Metagenomes and metatranscriptomes were pooled into groups based on chemical analysis and co-inertia results. Four groups were determined: early spring (ES) metagenomes, early spring (ES) metatranscriptomes, late spring (LS) metagenomes and late spring (LS) metatranscriptomes. Annotation diversity and differences in profiles between the genes retrieved in the metagenomes and the metatranscriptomes of these groups were compared with Venn diagrams using R package limma (Ritchie et al., 2015). Differential protein gene abundance was compared between the metagenomic profiles of the ES and LS groups using the R package edgeR (Robinson et al., 2010). The *p*-value was set at 0.05.

### Plasmid Marker and Antibiotic Gene Identification in Metagenomes and Metatranscriptomes

Plasmid structural related protein names were identified by retrieving the proteins annotated with the GO term id GO:0005727 (extrachromosomal circular DNA). In addition, a regular search of protein names using the keyword “plasmid” was carried out. Antibiotic response GO terms were extracted using a custom set of protein names retrieved from Uniprot (Supplementary Table S4 for complete list). Protein names annotated with the GO id GO:0017000 (antibiotic biosynthetic process) were also used. To mine for antibiotic resistance genes determinants (ARGDs) in both our metagenomic and metatranscriptomic datasets, reads were also annotated using Diamond blastx (Buchfink et al., 2015) against the CARD database (McArthur et al., 2013). All the hits that were returned with an *e*-value lower than 10^–10^, a *z*-score higher than 50 and a sequence similarity higher than 60% were considered as significant. For all the annotations, the best hit method was adopted to retrieve one unique annotation per read. Annotations were normalized by the total read count from their respective sample (after the removal of eukaryotic sequences from the total read counts).

## Results

### Snow Chemistry

**FIGURE 1:**
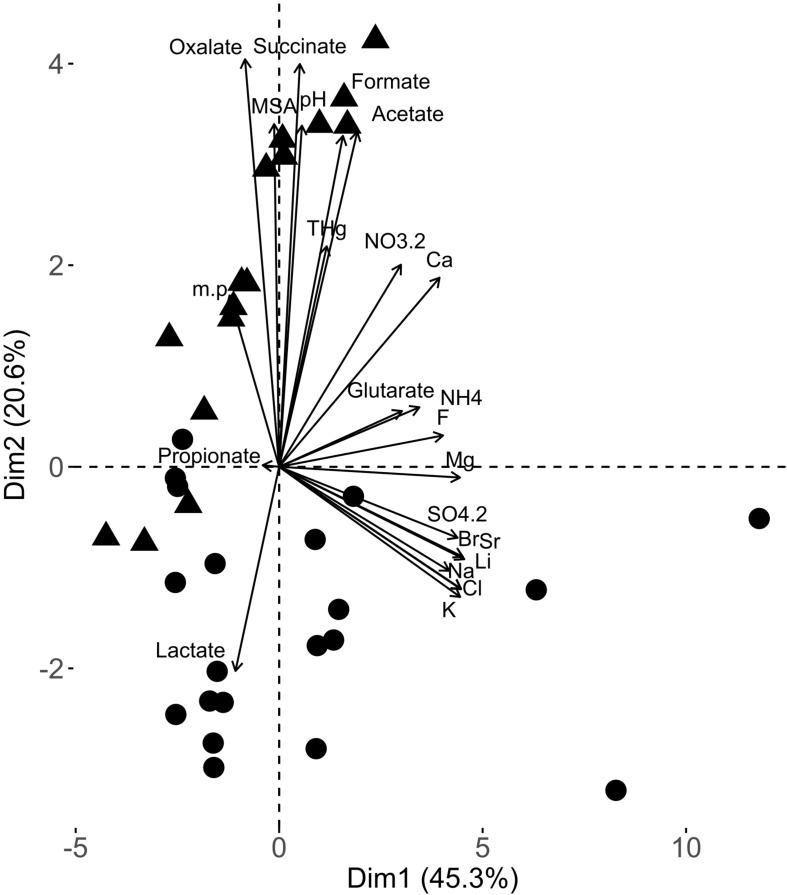
Principal component analysis biplot from the snow chemical analyses of the samples used in this study. The different chemical variables considered in this PCA are represented by vectors. The samples [black dots (early spring samples) and triangles (late spring samples)] are represented based on their respective projections.

Changes in snow chemical composition were monitored during the spring sampling period (April to June 2011, Supplementary Table S1). The chemical composition in early spring samples (ES) was different (PERMANOVA *p*-value = 0.0015) than late spring samples (LS) as shown by principal component analysis (PCA) (Figure 1). The difference in the early and late spring samples was due to the increase in most organic acids (acetate, oxalate, succinate and formate) and a decrease in lactate concentrations in late spring as well as changes in pH. Many inorganic salts (e.g., sulfate, bromide) were at higher concentrations in the early spring samples.

### Relationship Between Snow Chemistry and Microbial Data During Early and Late Spring

The co-variance of the chemistry and taxonomic datasets was determined (co-inertia coefficient RV = 0.48, *p*-value = 0.01). The co-inertia analysis did not highlight any clear relationship between taxonomy and chemistry. The metagenomic and metatranscriptomic relative abundances in different functional classes also co-varied with snow chemistry (Table 1). The level of annotation (i.e., proteins vs. gene onthology (GO) categories) influenced their relative co-variance. The co-inertia coefficient (RV) was the highest for metagenomic (vs. metatranscriptomic) datasets when using the GO terms. The co-inertia plot was similar to the PCA carried out using the chemistry data (Supplementary Figure S4). We observed a separation between the samples from the early and late spring along the first axis of the co-inertia plot (Supplementary Figure S4). The chemical variables with the highest influence on first axis of the co-structure were organic acids (acetate, succinate, oxalate and formate and lactate), pH and some major ions (fluoride, calcium). Similar to the OTU analysis, no specific proteins were found to have a significantly higher contribution to the co-inertia.

**TABLE 1:** Comparison of the different co-inertia calculated with the snow chemistry of the different snow samples and the different datasets such as 16S rRNA sequence clusters and the metagenomes/metatranscriptomes annotations determined with the Eggnog mapper.

**Dataset**	**Annotation**	**Co-inertia RV**	***p*-value**
Metagenomes	Genes id	0.44	0.033
	GO terms	0.45	0.003
	Kegg pathways	0.59	0.0002
Metatranscriptomes	Genes id	0.43	0.072
	GO terms	0.44	0.023
	Kegg pathways	0.37	0.064
16S rRNA sequencing	OTU 97% id	0.48	0.01

### Bacterial Community Structure

After filtering of the 16S rRNA gene reads, the samples had an average of 16 757 reads and a median of 8944 reads. Based on the annotation of cluster seeds using RDP classifier, the observed genera were mainly affiliated to Proteobacteria, Cyanobacteria, Bacteroidetes, Acidobacteria, Firmicutes and Actinobacteria. Linear correlation between individual variables was low (*R* = 0.14) and the analysis of similarity (ANOSIM) of the 16S rRNA gene derived OTUs from the early and late spring samples had a *p*-value = 0.03 (perm = 10 000). SIMPER analysis showed that the contribution from any individual OTU to the observed between-groups dissimilarity never exceeded 0.4%. The core community (defined as the OTUs appearing in more than 50% of the samples from one sampling period) from the early spring appeared to be bigger than the one from the late spring (59 vs. 29 OTUs with 17 shared OTUs between the two periods) (Supplementary Table S5). This threshold of 50% was based on the guidelines suggested by Weiss et al. (2016), although different levels up to 80% were examined and these higher values did not change the shared OTUs significantly. These two core communities (59 and 29 OTUs) were then used to build co-variance networks. The variations and annotation of the OTUs varied between samples and time during the spring season (Supplementary Figure S5).

### Exploring Cooperation Using Interaction Networks

**FIGURE 2:**
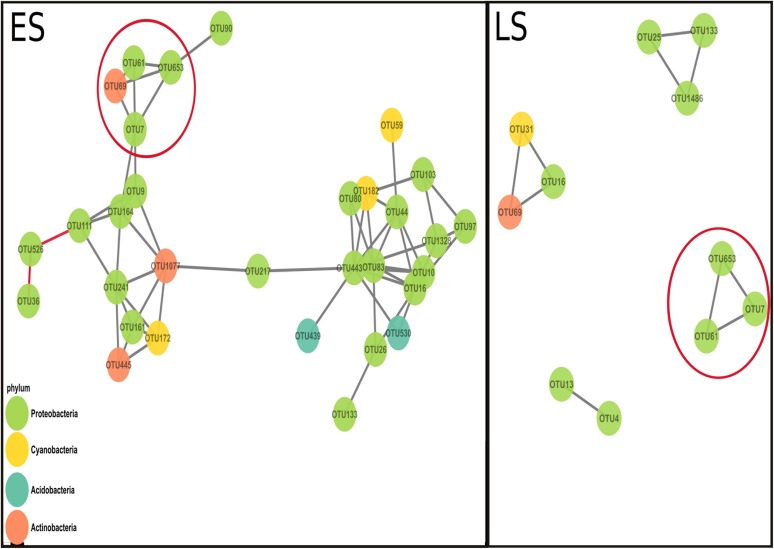
Co-variance networks built from the OTU normalized counts from early spring (ES) and late spring (LS). Each dot represents an OTU (the colors represent different phyla) and each black line represents a positive co-variance (considered as a surrogate of cooperation) and the two red lines in the ES networks represent two negative co-variances (interpreted as a possible competitive interaction). The red circles highlight the interactions that both networks shared. The average connectivity (average amount of positive co-variance a node possesses in a network) is higher in the ES network (=4) compared to the LS network (=1.82). The modularity was higher in the LS network (0.72) than in the ES network (0.532).

More OTUs co-varied positively in the early spring (ES) network than in the late spring (LS) network. The networks from early spring and late spring shared three interactions (Figure 2; red circles). The ES network displayed higher average node connectivity, but had a lower modularity than the network from the LS period (Table 2). The graph density and its transitivity were higher in the LS network, while the average edge betweenness and closeness were found to be higher in the ES network. We also investigated to which extend the size of the networks could be considered as different since the core communities from which the networks were derived were different in size (59 OTUs for ES vs. 29 OTUs for LS core community) (Supplementary Table S5). To do so, we considered the amount of interactions retrieved as positive in the respective networks (59 vs. 10) and standardized it by the total amount of possible interactions that were possible to build with their respective input sets of OTUs (i.e., which corresponds to a binomial coefficient computed for *n* = number of core OTUs and *k* = 2). This comparison confirmed our initial findings since the ratio of significant positive co-variances observed in each network was higher for ES (0.034) as compared to LS (0.025).

**TABLE 2:** The main network properties observed in the two co-variance networks build from OTU clusters of 16S rRNA gene sequencing data.

**Network property**	**Early spring**	**Late spring**
Average node connectivity	4	1.82
Modularity	0.532	0.72
Graph density (group adhesion)	0.14	0.18
Networks connectivity (group cohesion)	1	0
Transitivity	0.48	1
Average node closeness (normalized)	0.28	0.11
Average edge betweenness	36.62	0

### Bacterial Community Function

We used the KEGG metabolic pathways obtained from the EGGNOG annotations to determine the main metabolic pathways in the snow metagenomes and metatranscriptomes. The dominant pathways were similar for both metagenomes and metatranscriptomes (Supplementary Tables S6, S7) and were related to amino acid (i.e., arginine and proline metabolism), nucleic acid (i.e., purine/pyrimidine metabolism) and carbohydrate (butanoate, propionate and pyruvate) metabolism/catabolism. Nitrogen metabolism, bacterial chemotaxis, and ABC transporters were also present among the most abundant pathways. Pathways related to vitamin biosynthesis (i.e., folate *biosynthesis*), antibiotic metabolism (i.e., streptomycin and vancomycin biosynthesis pathways), methane metabolism, photosynthesis, cell motility (flagellar assembly), DNA repair, polyunsaturated fatty acid metabolisms as xenobiotic degradation (i.e., naphthalene, ketone) were also identified in the metagenomes and metatranscriptomes. Heatmaps with the 50 most abundant KEGG pathways in our metagenomes and metatranscriptomes are shown in Supplementary Figures S6, S7, respectively.

### Bacterial Community Functional Changes From Early (ES) to Late (LS) Spring

**FIGURE 3:**
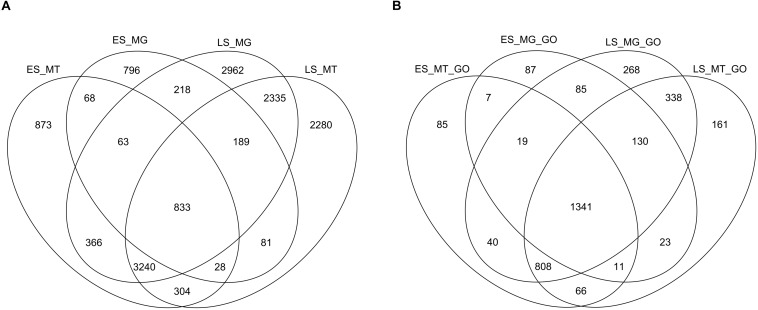
Venn diagrams displaying the functional overlap from the metagenomes (MG) and the metatranscriptomes (MT) from the early spring (ES) and the late spring (LS) periods based on two different levels of annotations (using EGGNOG-mapper) retrieved using UNIPROT: **(A)** protein name level and **(B)** GO (gene onthology) categories.

Venn diagrams were constructed at the protein level (gene product) and at the GO term level from the annotated metagenomic and metatranscriptomic datasets. At both the protein level and the GO level, a more diverse group of genes was annotated for LS samples than for ES samples (Figure 3). The metagenomes and metatranscriptomes in late spring shared more genes between them than they did in early spring. In addition, the overlap between early spring metatranscriptomes and late spring metagenomes was larger than the overlap between early spring metagenomes and early spring metatranscriptomes. The overlap between early spring and late spring metatranscriptomes was larger than the overlap between early spring metatranscriptomes and metagenomes.

The GO categories that were more abundant in ES metagenomes and metatranscriptomes were related to resistance to chloramphenicol, plasmid maintenance, and cellular stress like ribophagy and autophagy (see Supplementary Table S8 for details). Among the GO categories that were more abundant in LS metagenomes and metatranscriptomes, several were related to lactate/oxalate catabolism and acetate and formate metabolism as well as phosphate starvation (see Supplementary Table S9 for details). Some examples for acetate include cation/acetate symporter (log FC 4.4, *p*-value 0.004) and acetyl-coenzyme A synthetase (logFC 4.1, *p*-value 0.001). The proteins names retrieved as being in relation with organic acid catabolism were formyl-CoA:oxalate CoA-transferase (FCOCT) and formate dehydrogenase (FDH). The tax ids from those genes were from bacterial species from the Comamonadaceae and the Ralstoniaceae, two families from the order of Burkholderiales. Virus related terms (i.e., viral process and capsule organization) were also more abundant in the late spring samples.

**FIGURE 4:**
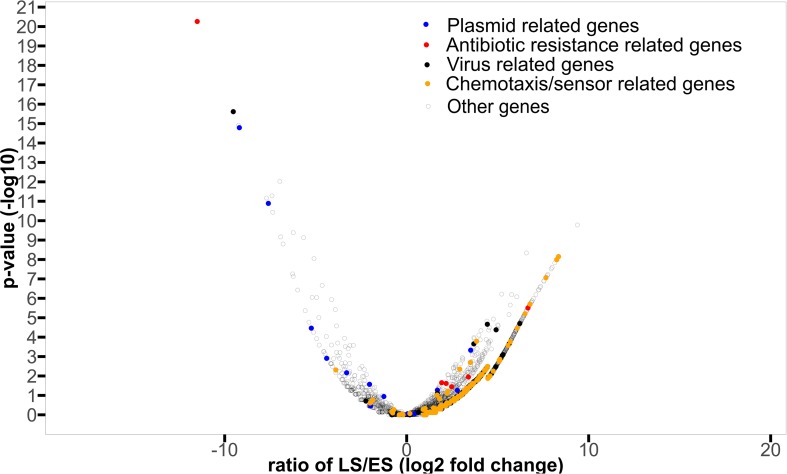
Volcano plot displaying the protein names significantly enriched in early or late spring metagenomes compared to the other period. The log10 of the *p*-value significance of the differential abundance study retrieved from edgeR is plotted as a function of the logFold change observed for the respective protein names used in the study (filtered out for occurrences lower than two samples). The cutoff of *p*-val > 0.05 (log10(0.05) = 1.3) has been used in this study. The plasmid structural protein names (replication proteins and toxin anti-toxin complex, considered as surrogate of bacterial cooperation) identified are plotted as blue dots, the antibiotic resistance/synthesis protein names (surrogate of bacterial competition) are plotted as red dot. We plotted protein names related to viruses in black and protein names related to chemotaxis and sensors as orange dots.

In total, 1463 proteins were shown to be significantly more abundant in the metagenomic dataset from either of the two sampling periods by EdgeR (see Supplementary Table S10 for more abundant in ES and Supplementary Table S11 for more abundant in LS for details) of which 125 were more abundant in ES metagenomes (logfold < 0), while 1338 were more abundant in LS metagenomes (logfold > 0) (Figure 4). The annotated proteins that were most enriched in the ES metagenomes with the largest logfold changes between early and late spring were linked to chloramphenicol resistance (logFold = −11.5), plasmid structure genes (logFold = −7.6) (Supplementary Table S10 for details). Annotated proteins involved in plasmid maintenance and plasmid partition were more abundant in the early spring (Table 3). Annotated proteins that had the largest logfold changes between late and early spring were linked to environmental sensing (logFold = 7.6–8.3), a membrane-transport protein (logFold = 8.1) and a putative exported protein (logFold = 9.4). Antibiotic resistance proteins (tetR, penicillin binding protein, bleomycin resistance, and macrolide resistance) and proteins involved in antibiotic biosynthesis (amidase) were more abundant in the late spring (Table 4). Sequences related to viruses and chemotaxis were also observed at higher abundances in LS metagenomes (Supplementary Table S11 for details).

**TABLE 3:** Protein names related to plasmid structure genes determined by edgeR as being significantly enriched in metagenomes from early spring (logFC < 0) or late spring (logFC > 0).

**Protein**	**logFC**	**logCPM**	***P*-value**
Replication initiation protein (Protein E) (Protein rep)	−9.193	12.645	1.62 × 10^–15^
Rep protein (Fragment)	−7.600	11.182	1.288 × 10^–11^
Putative plasmid maintenance system antidote protein, XRE family	−5.240	9.607	3 × 10^–5^
XRE family plasmid maintenance system antidote protein	−4.397	9.255	0.001
Plasmid maintenance system killer	−3.300	9.107	0.007
Plasmid recombination protein	−2.041	10.565	0.027
Plasmid recombination protein.1	−2.041	10.565	0.027
Replication protein	3.517	11.551	0.0005

*The logCPM represents the average abundance of the protein name across the whole dataset and is an indicator of how much signal was present in the dataset to test the enrichment with edgeR.*

**TABLE 4:** Protein names related to antibiotic resistance or synthesis genes returned by edgeR as being significantly enriched in metagenomes from early spring (logFC < 0) or late spring (logFC > 0).

**Protein**	**logFC**	**logCPM**	***P*-value**
Chloramphenicol acetyltransferase (EC 2.3.1.28)	−11.509	14.915	5.50 × 10^–21^
Transcriptional regulator, TetR family	1.922	11.399	2.2 × 10^–2^
Beta-lactamase	2.168	10.564	2.4 × 10^–2^
Penicillin-binding protein 1B (PBP-1b) (PBP1b) (Murein polymerase)	2.471	9.616	0.036
Penicillin-binding protein 2	3.301	9.189	0.043
Glyoxalase/bleomycin resistance protein/dioxygenase	3.393	9.801	0.011
Macrolide export ATP-binding/permease protein MacB (EC 3.6.3.-)	3.807	9.312	0.017
Penicillin-binding protein	3.873	9.338	0.017
Putative amidase	6.663	10.997	3.15e – 06

*The logCPM represents the log2 average abundance of the protein name across the whole dataset and is an indicator of how much signal was present in the dataset to test the enrichment with edgeR.*

### Changes in Antibiotic Resistance Gene Determinants in the Snow

**FIGURE 5:**
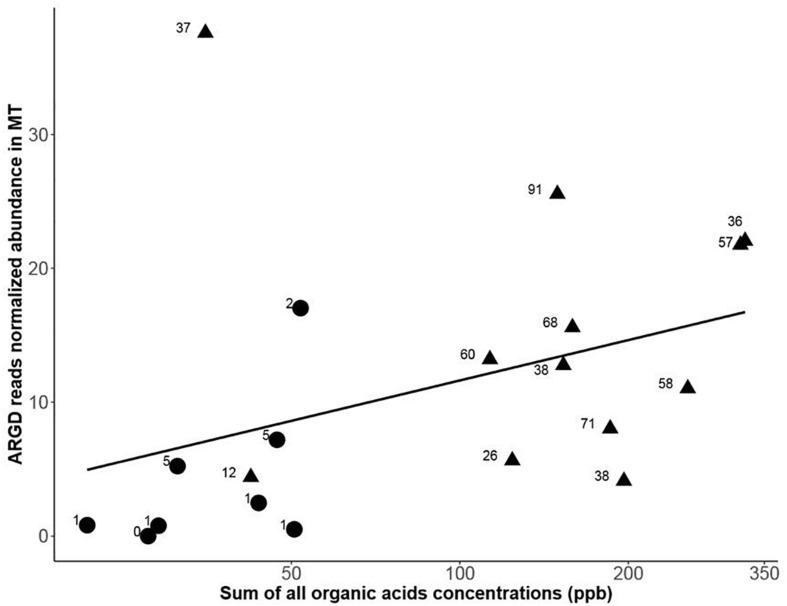
Antibiotic resistance genes (ARGD) transcription annotated from the metatranscriptome datasets (MT) vs. the total sum of organic acids amounts measured in the snow samples (black dot, early spring samples and black triangle, late spring samples). The numbers display the amount of different ARGD genes annotated in each sample. A Spearman correlation between ARGD transcription and total organic acids concentration had a rho = 0.57 and a *p*-value = 0.010.

Using the CARD antibiotic resistance gene database (McArthur et al., 2013), metagenomic and metatranscriptomic sequences were annotated for antibiotic resistance genes. The number of the different antibiotic resistance gene determinants (ARGDs) was greater for the late spring samples and the overlap between metagenomes and metatranscriptome ARGDs was higher for the late spring samples (Supplementary Figure S8). Both the number of metatranscriptomic sequences annotated as ARGDs and the diversity of these genes correlated to organic acid concentrations (Figure 5). The annotated early spring taxonomy of the chloramphenicol acetyl-transferase had two tax ids from the database (*Clostridium scindens and Pseudoflavonifractor capillosus*). For the late spring samples, the sequences annotated as the putative amidase were assigned eight different taxa ids (two strains of *Pseudomonas fluorescens, Nocardia farcinica, Gemmatimonas aurantiaca, Sinorhizobium fredii, Rubrivivax benzoatilyticus*, and *Gordonia alkanivorans*). The sequences annotated as the protein MacB involved in macrolide resistance was assigned to four different tax ids (*P. fluorescens, Stenotrophomonas maltophilia, Nostoc* sp., and *Achromobacter insuavis*). Interestingly, *Pseudomonas* was found in the early spring interaction network and implicated in a negative interaction.

## Discussion

### Interactions Between Organic Acids and Bacterial Communities in Snow

Among the different snow chemical parameters that were tightly coupled to changes in microbial functions (metagenomic) (Table 1), total measured organic acid concentration ranged from around 3 ppb to over 2000 ppb (see Supplementary Table S1). For samples that had metagenomic sequencing performed, the total organic acids ranged from 6ppb to 350 ppb (see Figure 5). Increases in organic acid concentrations were previously observed in Svalbard (Larose et al., 2013) and Greenland snow (Twickler et al., 1986). We also saw an increase in genes related to organic acid metabolism (e.g., acetate catabolism) in LS metatranscriptomes, which could reflect an active response of the snow community. Metatranscriptomes might provide a sensitive and rapid indicator of environmental signals while metagenomes might be more representative of changes over longer periods of time in relation to their chemical environment. The late spring protein-coding genes (both from metagenomic and metatranscriptomic data sets) overlapped more with the metatranscriptomes from the early spring than with the metagenomes from the early spring (Figure 3). These trends were also observed at the GO term annotation level. This pattern might indicate that some of the low abundance active taxa from the early spring season (not observed in the metagenomes but observed in the metatranscriptomes from early spring) became dominant during the late spring season (observed in the late spring metagenomes) and stayed active during this period (also present in late spring metatranscriptomes). This was consistent with the associated taxonomy based on the functional gene annotation where taxa observed only in the early metatranscriptomes but not in the metagenomes that were also retrieved in the late spring metagenomes and metatranscriptomes (Supplementary Figure S9).

### Bacterial Communities of the Snow Shift From Cooperation Toward Competition as Organic Acid Levels Increased

Plasmids might be involved in cooperative interactions and could serve as a marker for microbial collaboration. For example, genes coding for public goods were preferentially located on mobile elements or close to integrases when incorporated into genomes (Nogueira et al., 2009; Mc Ginty et al., 2011). In addition, conjugation and gene transfer through plasmids were associated with bacterial cooperation (Dimitriu et al., 2014). Gene transfer might drive cooperation among bacteria by increasing their genetic similarity that would select cooperative behavior via kin selection (Nogueira et al., 2009). The sequences related to plasmid structural proteins were more abundant in early spring metagenomes than in late spring when the organic acid concentrations were higher. While this does not show causality, it is consistent with the hypothesis that organic acids might impact microbial interactions.

Antibiotics might be proxies for bacterial competition and their related marker genes (production and resistance) have been used to track bacterial interference competition (Ponce-Soto et al., 2015; Goordial et al., 2017). In our study, sequences annotated as antibiotic resistance and secretion proteins were more abundant in late spring metatranscriptomes and metagenomes (Figure 4). Sequences annotated as putative amidase, penicillin amidase, and penicillin amylase were only observed in the late spring metagenomes. These proteins are known to be involved in some derivatives of penicillin and lactone biosynthesis; this last molecule is one of the main constituents of macrolide antibiotics (Omura, 2002). We correlated an increase in the number and diversity of antibiotic resistant gene determinants to an increase of organic acid content in the snow (Figure 5). Competition might increase as the environment becomes richer in organic acids and result in bacterial communities actively transcribing genes for an increasingly diverse set of ARGDs. While antibiotic resistance is also sometimes associated with cooperative traits (Cordero et al., 2012), the diversity of antibiotic genes would be low as the entire community shares the public good. In our data sets, only early spring samples had low antibiotic gene diversity (see Table 4), which might be compatible with the hypothesis of antibiotics secreted as a public good to protect the whole cooperative community.

Physical changes of the snowpack might also induce a shift from cooperation to competition. As the season progressed, the snowpack became gradually warmer and wetter. This likely increased motility of the bacterial population within the snow as indicated by an increase in the relative abundance of proteins related to chemotaxis and motility (i.e., receptors, flagella) in late spring samples (Supplementary Tables S9, S10). A decrease in the environmental stratification of the snow ecosystem with observed changes in snow crystal morphology (from faceted crystals to rounded ones) and a loss of snow layers was also apparent throughout the entire spring period. Several studies have shown that bacterial cooperation was counter-selected when the stratification of the environment decreased to the benefit of competitive bacterial strains (F. J. H. Kümmerli et al., 2009; Hol et al., 2013, 2015). The transition from a cold dry snowpack to a warmer wetter one might have led to increased habitat mixing among micro-organisms. Increased mixing could increase the viral-microbial contact, which would lead to increased infection rate (Ashby et al., 2014; Simmons et al., 2018). This possible increased infection rate was consistent with the increased viral related sequences and GO terms in late spring metagenomes (Supplementary Tables S9, S11 for details).

### Microbial Networks Respond to the Shift of Cooperation Toward Competition

Co-variance networks have been used recently to study bacterial interactions and two network characteristics, connectivity and modularity, were considered as proxies for cooperation and competition, respectively. The early spring (ES) network had a higher average connectivity than the late spring network (Figure 2 and Table 2). This was further confirmed by the higher ratio observed between the positive interactions retrieved in the ES network and all the possible interactions than the same ratio for the LS network. We also compared the intensity of the respective co-variances observed in these two networks by looking at their respective local spatial autocorrelation (LSA) coefficients (similar to a correlation coefficient with values between 0 and 1 for positive co-variances) and did not observe any significant differences in their distribution [between 0.81 and 0.91 (Supplementary Figure S10)]. Higher average connectivity can be interpreted as a marker of cooperation within the early spring bacterial community. This property is also related to an increased resistance to change (local resilience) since the presence of several organisms within the network can contribute to resisting to local perturbations (Scheffer et al., 2012). In the context of positive bacterial interactions, metabolic exchanges between the different members of the community could enhance the resilience of the cooperative strains when the nutrient composition changes. As shown by Benomar et al. (2015), nutrient stress can induce metabolic exchanges between two bacterial strains. However, once perturbations are too great, the whole network structure can be transformed (Scheffer et al., 2012). The overlap between the covariance networks of early and late spring communities was low (only two interactions, see Figure 2), even though their core communities overlapped by more than 50% of the OTUs (Supplementary Table S5). The changes in nutrients over a short period of time and the decrease in environmental stratification might have led to the differences in the positive interaction networks for the bacterial communities from early and late spring snow (Figure 2 and Table 2).

The late spring network displayed a higher modularity than the early spring network. High modularity is linked to a higher adaptive capacity, since the network is more heterogeneous (Scheffer et al., 2012). This network configuration could be more advantageous in a dynamic environment where perturbations are more intense. An increase in environmental perturbations has also been associated with a decline in cooperation (Wilson et al., 2017). This effect was explained by a trade-off between access to nutrients (enhanced by spatial perturbations) and access to an auto-inducer to initiate cooperation (decreased by spatial perturbations) (Wilson et al., 2017). In our data, we observed more GO terms related to stress (mainly due to antibiotics and viruses but also to oxidative and osmotic stress) in the late spring metagenomes relative to the early spring metagenomes.

## Conclusion

Increase in organic acid concentrations in the snow might have influenced bacterial interactions and led to a shift from cooperation to competition. Several other correlations were observed between community response and environmental chemical parameters. Physical changes of the snow structure leading to decreased stratification and increased mixing might have also contributed. Using a combined method of marker genes and network analysis, we evaluated bacterial interactions in the complex snow microbial communities. Future work should include controlled laboratory studies with snow enriched with organic acids to confirm the trends observed in this field study. In addition, we need to increase our knowledge of genetic markers of microbial interactions since the number of genes currently used to track cooperation and competition is still small and controversial.

## Data Availability Statement

Sequences generated are publically available at ftp://ftp-adn.ec-lyon.fr/Snow_organic_acids_bacterial_interactions.

## Author Contributions

BB, CL, AD, and TV conceived the experiment. BB performed the lab work and all the bioinformatics analyses. LM performed the lab work and method development. AD and CL were involved in setting up the sampling. AD performed the chemical analyses. All the authors were involved in writing and reviewing the manuscript.

## Conflict of Interest

The authors declare that the research was conducted in the absence of any commercial or financial relationships that could be construed as a potential conflict of interest.

## Footnotes

1 http://www.uniprot.org/uploadlists/
