# Supplementary material for: Do Organic Substrates Drive Microbial Community Interactions in Arctic Snow?
Source: Front Microbiol. 2019 Oct 31;10:2492. doi: 10.3389/fmicb.2019.02492 (PMC6842950; doi:10.3389/fmicb.2019.02492)
Supplement: Supplementary file 12 [file Data_Sheet_1.PDF]

Supplementary Material

1 Supplementary Data

SI_clustering_UPARSE.docx: Additional information concerning the parameters used to process the 16S rRNA gene amplicon sequencing data.

UPARSE_snow.sh: Script used to cluster the 16S rRNA gene amplicon sequencing data.

2 Supplementary Figures and Tables

2.1 Supplementary Figures

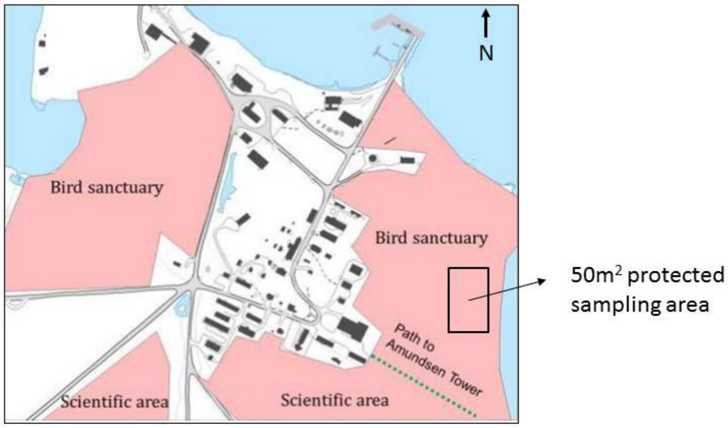

Figure S1: Overview of the sampling area. This image was modified from a document available on the Kingsbay website (https://kingsbay.no/visitor_information/content/textwithimage_3eddc5cb-0349-4f82-866c-47435ead9eeb/1486385662646/info_ny__eng_2016_brochure.pdf)

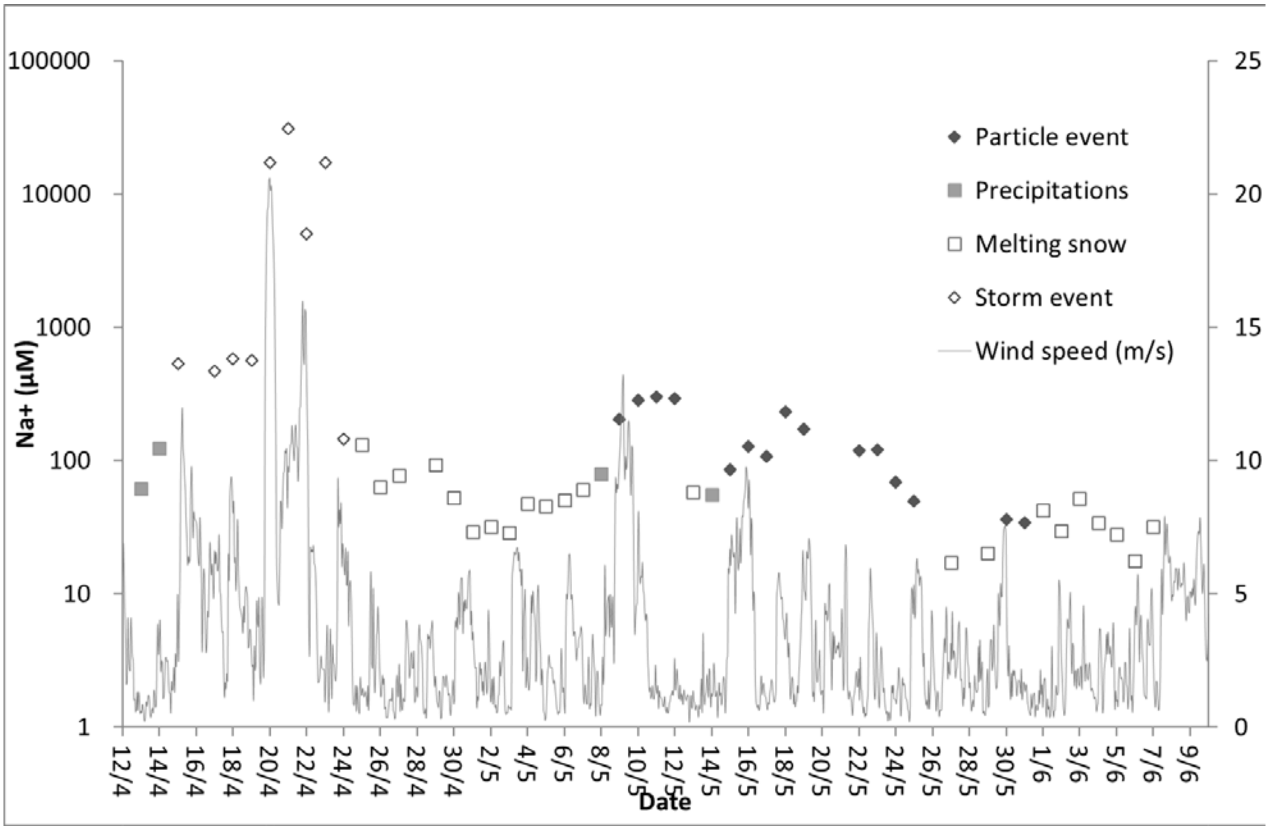

Figure S2: Overview of changes in snowpack characteristics and weather events throughout the sampling period. Individual points refer the sodium concentrations as a marker for possible seawater exposure. These points are separated into open diamonds for storm events (characterized by high winds and changes in wind direction), grey squares for precipitation events (snowfall), black diamonds for particle deposition events and open squares for surface melting events. Sodium concentrations for each microbial sampling time are shown in Table S1.

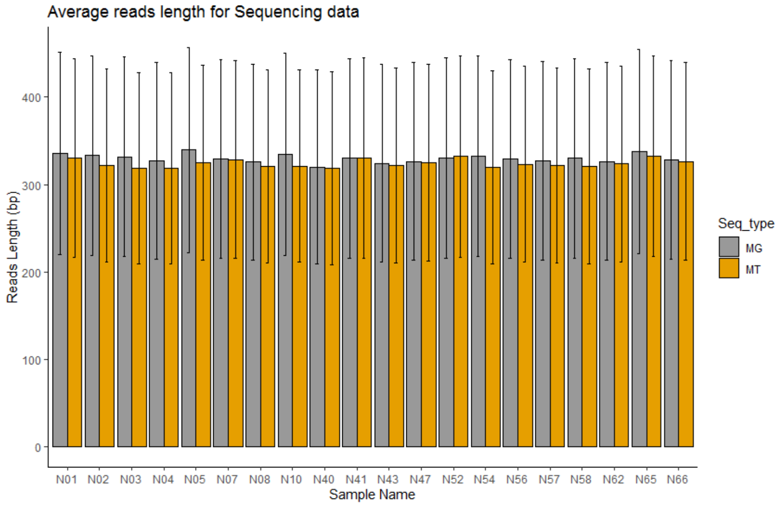

Figure S3: Bar plot of the average read length of the metagenomic and metatranscriptomic pyrosequenced samples following quality filtering and trimming. Error bars represent the standard deviation.

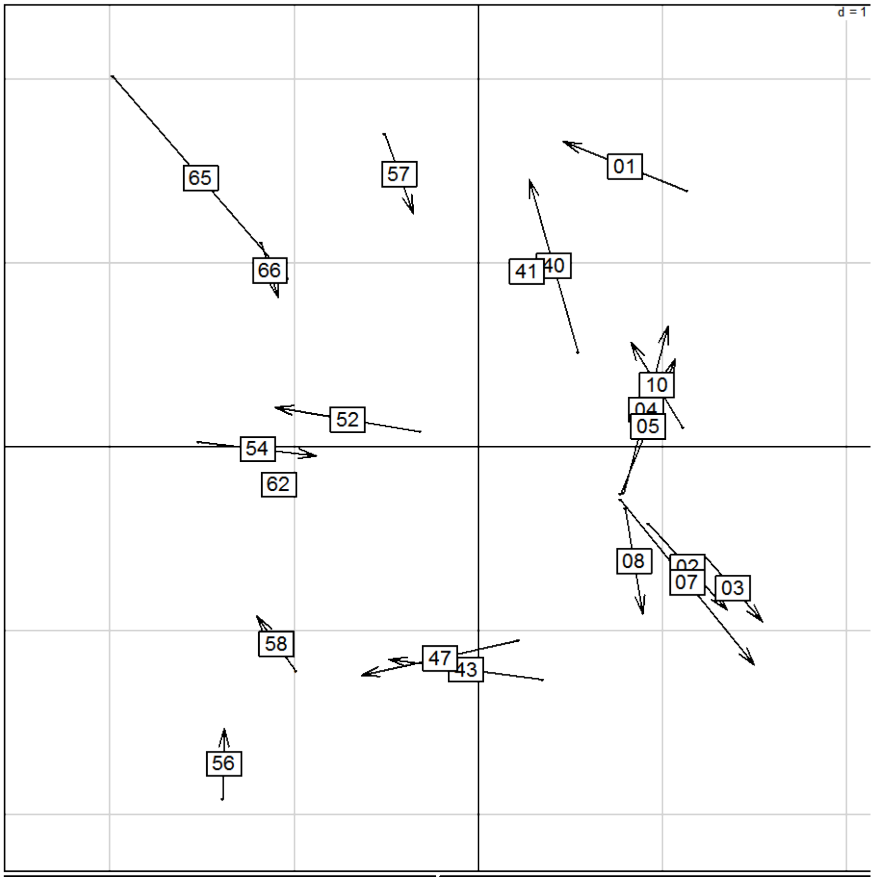

Figure S4: Co-inertia output graph for the protein names from metagenomes versus the environmental data generated by ade4 R package. Samples with IDs below 40 represent early spring samples and those from 40 and above represent late spring samples. Sample numbers are also listed in Table S1 as CH3N-1 through CH3N66. Arrows represent co-variance scores between the relative

GO term reads and environmental data. The shorter the arrow, the higher the correlation between data sets (metagenome and environmental variables) for each sample.

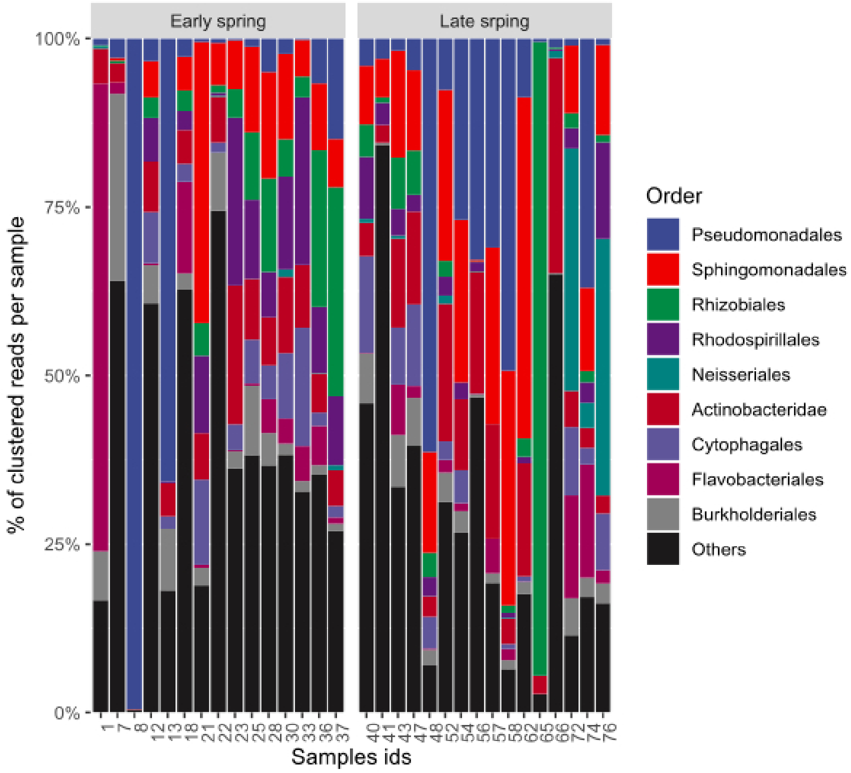

Figure S5: Histogram representing OTU distribution across samples at the order level. Samples with IDs below 40 represent early spring samples and those from 40 and above represent late spring samples. Sample numbers are also listed in Table S1 as CH3N-1 through CH3N76.

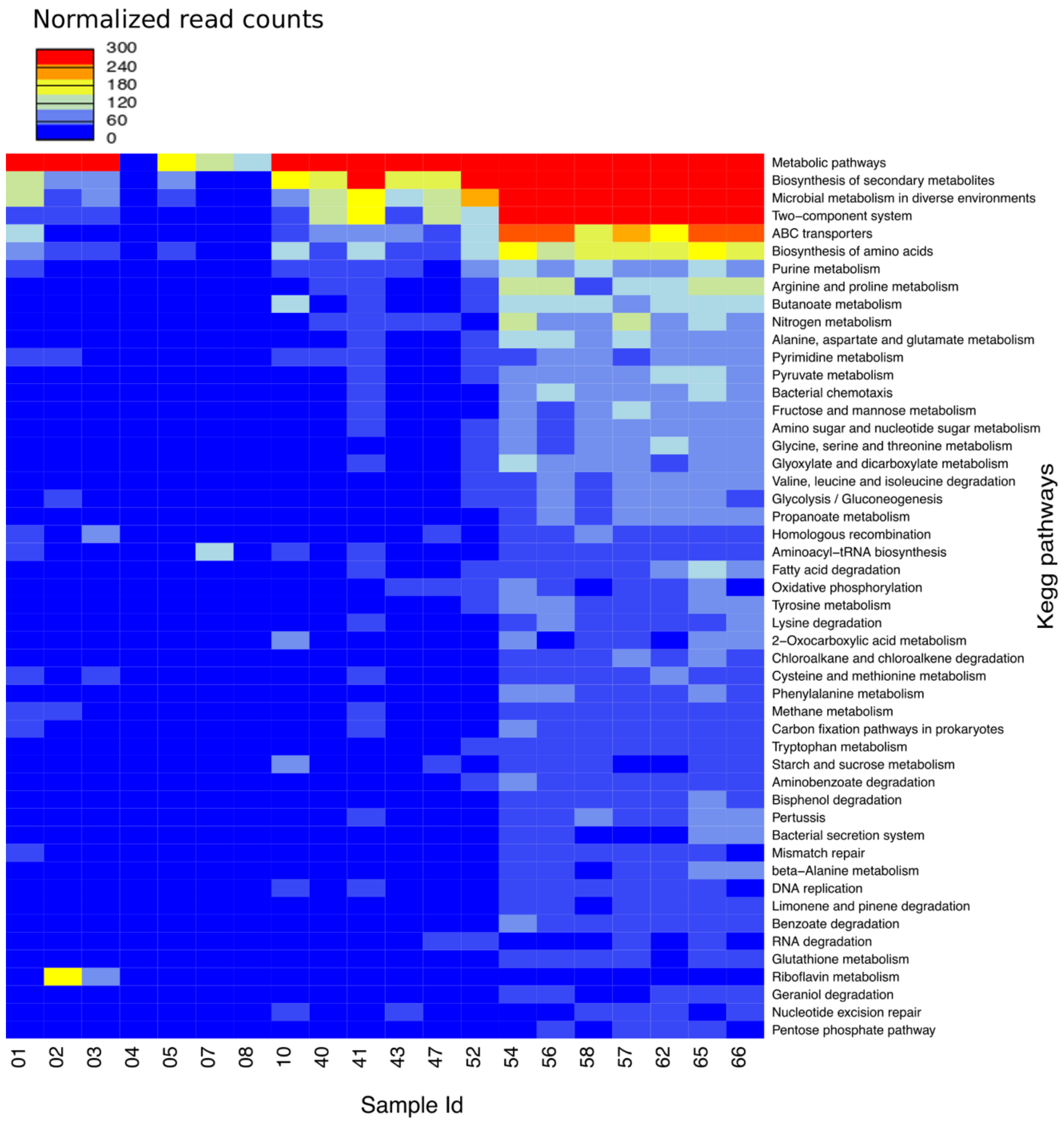

Figure S6: Heatmap displaying the 50 most abundant Kegg pathways observed in the metagenomes from this study. Samples with IDs below 40 represent early spring samples and those from 40 and above represent late spring samples. Sample numbers are also listed in Table S1 as CH3N-1 through CH3N66.

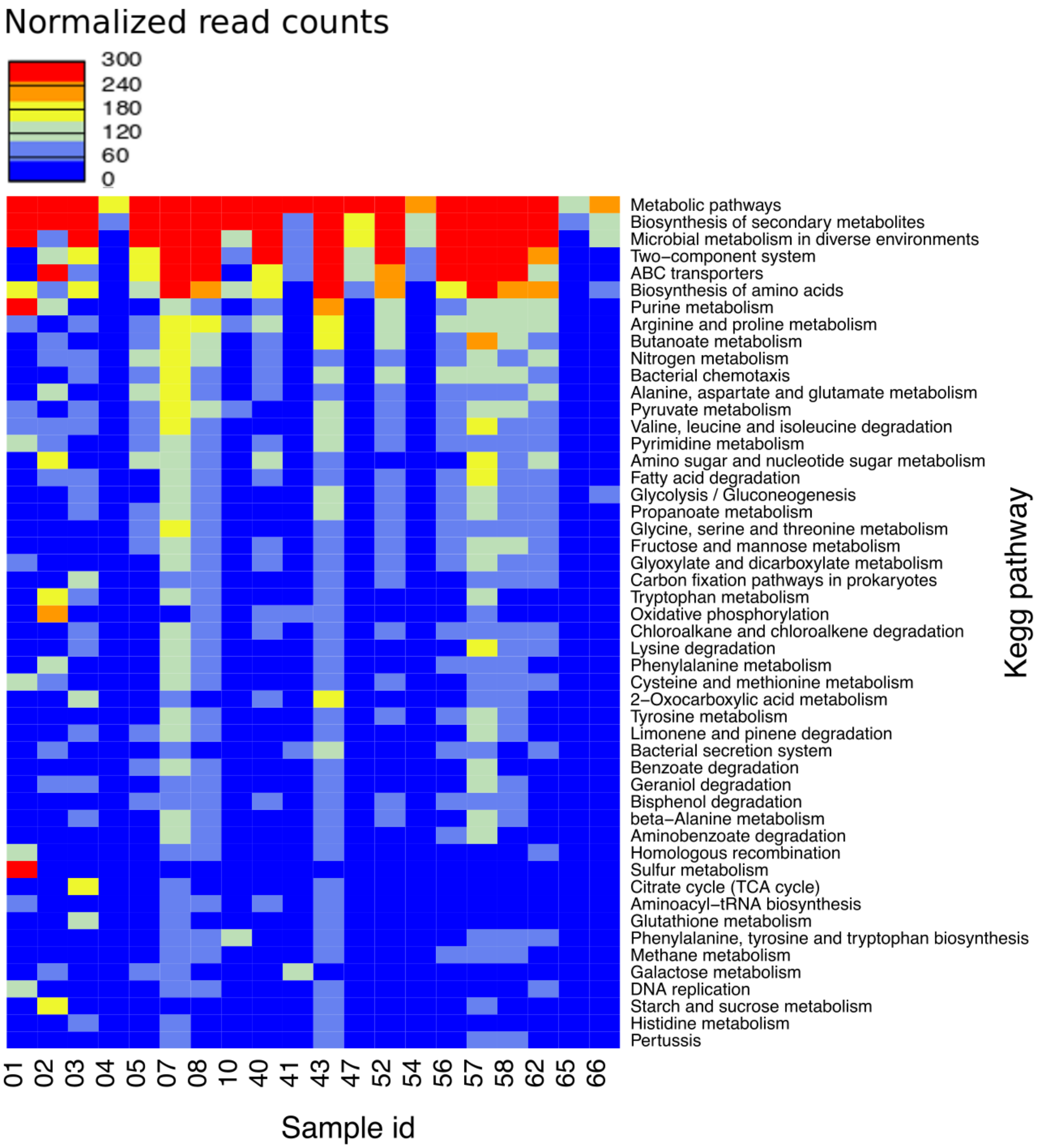

Figure S7: Heatmap displaying the 50 most abundant Kegg pathways observed in the metatranscriptomes from this study. Samples with IDs below 40 represent early spring samples and those from 40 and above represent late spring samples.

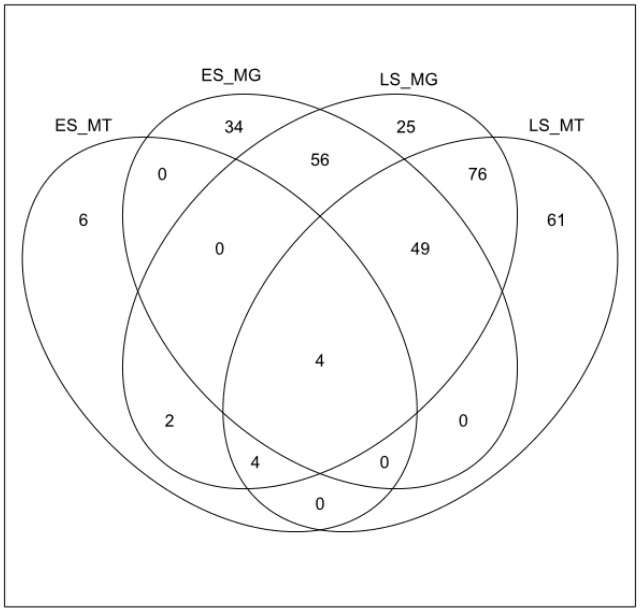

Figure S8: Venn diagram summarizing the different annotated ARGDs shared between metagenomes (MG) and metatranscriptomes (MT) from the samples collected during the early (ES) and late (LS) spring.

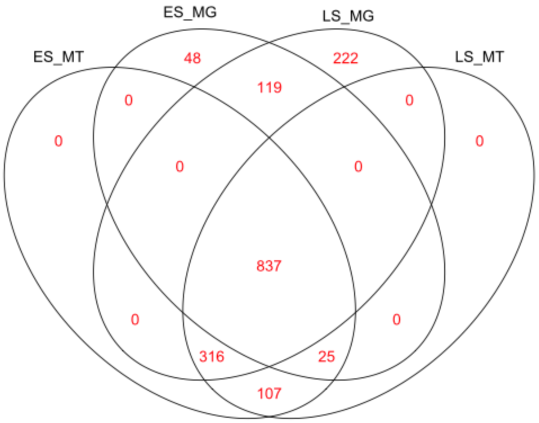

Figure S9: Venn diagram summarizing the overlaps between the taxonomic annotations retrieved from metagenomes (MG) or metatranscriptomes (MT) from the early spring (ES) and late spring (LS) samples.

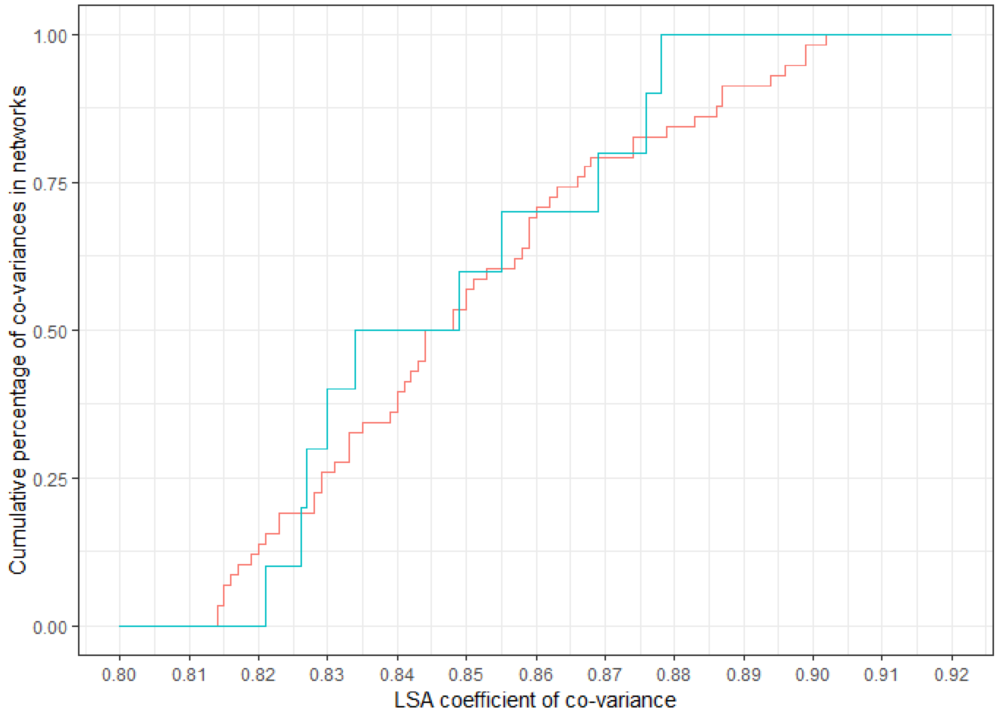

Figure S10: Cumulative curves showing the number of co-variances returned by fastLSA in the ES network (red) and in the LS network (blue).

2.2 Supplementary Tables

Table S1 (Table_S1.xlsx) Table providing all the chemistry measurements carried out on the different samples from the study. In addition, two columns summarize which samples were sequenced in metagenomic/metatranscriptomic (454 pyrosequencing) and 16S rRNA gene (miSeq paired read sequencing). Chemical measurements below detection limit are reported as '<DL' and missing measures as 'NA'.

Table S2 (Table_S2.xlsx) Table summarizing the initial amount of sequences (sum of forward and reverse reads) for each 16S rRNA gene sequencing and how many were successfully clustered by Usearch into OTUs.

Table S3 (Table_S3.xlsx) Table summarizing the amount of sequences per sample after quality filtering and how many of them where successfully annotated using eggnog-mapper.

Table S4 (Table_S4.xlsx) Protein names involved in antibiotic resistance retrieved from uniport.

Table S5 (Table_S5.xlsx) Table summarizing the different OTUs remaining in early spring (EScore) and late spring (LScore) communities after filtering out OTUs that were not present in at least eight samples. The core community from the early spring was larger (59 OTUs) than the core community from the late spring (29 OTUs). Their respective core communities overlapped by 17 OTUs.

Table S6 (Table_S6.xlsx) Table of the 100 most abundant kegg pathways in the metagenomes from both sampling periods based on the average number of annotated reads.

Table S7 (Table_S7.xlsx) Table of the 100 most abundant kegg pathways in the metatranscriptomes from both sampling periods based on the average number of annotated reads.

Table S8 (Table_S8.html) Table of the GO terms retrieved exclusively in metagenomes and/or metatranscriptomes from the early spring period.

Table S9 (Table_S9.html) Table of the GO terms retrieved exclusively in metagenomes and/or metatranscriptomes from the late spring period.

Table S10 (Table_S10.xlsx) Protein names determined by edgeR as being significantly enriched in metagenomes from early spring (logFC<0). The logCPM represents the average abundance of the protein name across the whole dataset and is an indicator of how much signal was present in the dataset to test the enrichment with edgeR.

Table S11 (Table_S11.xlsx) Protein names determined by edgeR as being significantly enriched in metagenomes from late spring (logFC>0). The logCPM represents the average abundance of the protein name across the whole dataset and is an indicator of how much signal was present in the dataset to test the enrichment with edgeR.
